# Psychobehavioral Profiles to Assist Tailoring of Interventions for Patients With Hypertension: Latent Profile Analysis

**DOI:** 10.2196/jmir.8757

**Published:** 2018-05-11

**Authors:** Rika Tanaka, Robert P Nolan

**Affiliations:** ^1^ Cardiac eHealth and Behavioural Cardiology Research Unit Peter Munk Cardiac Centre University Health Network Toronto, ON Canada; ^2^ Department of Psychiatry and Institute of Medical Sciences Faculty of Medicine University of Toronto Toronto, ON Canada

**Keywords:** hypertension, depression, health behavior, lifestyle, counseling

## Abstract

**Background:**

Practice guidelines advocate combining pharmacotherapy with lifestyle counseling for patients with hypertension. To allow for appropriate tailoring of interventions to meet individual patient needs, a comprehensive understanding of baseline patient characteristics is essential. However, few studies have empirically assessed behavioral profiles of hypertensive patients in Web-based lifestyle counseling programs.

**Objective:**

The objectives of this study were to (1) specify baseline psychobehavioral profiles of patients with hypertension who were enrolled in a Web-based lifestyle counseling trial, and (2) examine mean differences among the identified profile groups in demographics, psychological distress, self-reported self-care behaviors, physiological outcomes, and program engagement to determine prognostic implications.

**Methods:**

Participants (N=264; mean age 57.5 years; 154/264, 58.3% female; 193/264, 73.1% white) were recruited into a longitudinal, double-blind, randomized controlled trial, designed to evaluate an online lifestyle intervention for hypertensive patients. A series of latent profile analyses identified psychobehavioral profiles, indicated by baseline measures of mood, motivation, and health behaviors. Mean differences between profile groups were then explored.

**Results:**

A 2-class solution provided the best model fit (the Bayesian information criterion (BIC) is 10,133.11; sample-size adjusted BIC is 10,006.54; Lo-Mendell-Rubin likelihood ratio test is 65.56, P=.001). The 2 profile groups were (1) adaptive adjustment, marked by low distress, high motivation, and somewhat satisfactory engagement in health behaviors and (2) affectively distressed, marked by clinically significant distress. At baseline, on average, affectively distressed patients had lower income, higher body mass index, and endorsed higher stress compared with their adaptive adjustment counterparts. At 12-months post intervention, treatment effects were sustained for systolic blood pressure and Framingham risk index in the adaptive adjustment group, and those in the adaptive adjustment group were 2.4 times more likely to complete the 12-month intervention study, compared with their affectively distressed counterparts.

**Conclusions:**

Interventions for patients who are adaptively adjusted may differ in focus from those designed for the affectively distressed patients. As such, this study underscores the importance of identifying psychobehavioral profiles, as they allow for evidence-based tailoring of lifestyle counseling programs for patients with hypertension.

**Trial Registration:**

ClinicalTrials.gov NCT01541540; https://clinicaltrials.gov/ct2/show/NCT01541540 (Archived by WebCite at http://www.webcitation.org/6yzZYZcWF)

## Introduction

### Background

Elevated systolic blood pressure (SBP) places individuals at increased risk of cardiovascular disease (CVD), stroke, coronary heart disease, heart failure, and CVD mortality [1]. Nevertheless, blood pressure is treated and controlled in only two-thirds of those diagnosed with hypertension [2,3], leaving a large proportion of this population at significant risk for the development of further cardiac risk. Current guidelines consider the combination of pharmacotherapy with lifestyle counseling as best practice for the management of hypertension [4,5].

As the feasibility and clinical utility of large-scale motivational interviewing and cognitive behavioral therapy-based lifestyle counseling programs continue to be established and further disseminated for patients with hypertension [6-10], it has become increasingly important to develop a systematic, comprehensive, and efficient way to assess the psychological and behavioral characteristics of the growing number of patients enrolling in such programs. A comprehensive understanding of baseline patient characteristics is essential to allow for more appropriate tailoring of interventions to meet individual patient needs. In keeping with this objective, psychometric instruments have been developed to identify adaptive and maladaptive patterns of adjustment among patients with chronic pain, and preliminary work has been reported for patients with cardiovascular conditions [11,12]. Few studies have empirically assessed psychological or behavioral profiles of hypertensive patients enrolled in Web-based lifestyle counseling programs.

### Objectives

The goals of this study were to (1) assess and specify baseline psychobehavioral profiles of patients with hypertension who were enrolled in a Web-based lifestyle counseling trial, (2) examine mean baseline differences among the identified profile groups in demographics, psychological distress, and self-reported self-care behaviors, and (3) assess differences in physiological outcomes (SBP; diastolic blood pressure, DBP; pulse pressure, PP; and Framingham risk index, FRI) and program engagement across these profile groups over 12 months to determine prognostic implications.

## Methods

### Overview

This is a substudy of the Reducing risk with E-based support for Adherence to lifestyle Change in Hypertension (REACH) trial [13], which was a multicenter, longitudinal, double-blind, randomized controlled trial of e-counseling for persons with stage 1 or 2 hypertension (Clinicaltrial.gov: NCT01541540). REACH was designed to evaluate the efficacy of a standardized, evidence-based e-counseling protocol that promoted adherence to recommended guidelines for exercise, diet, prescribed medications, and smoke-free living over 12 months. In the parent study, eligible hypertensive participants were recruited across 5 Canadian sites: Toronto (n=174), Vancouver (n=39), Grey Bruce (n=19), London (n=15), and Prince Edward Island (n=17). All participants were randomly assigned to treatment or control interventions after their eligibility was confirmed at their baseline visit. Primary endpoints of the REACH study included SBP and DBP, PP, non-high-density lipoprotein cholesterol, and the FRI of 10-year absolute risk of CVD.

### Study Interventions and Assessments

Both the control and e-counseling arms of REACH were organized by sessions that included a URL that linked participants to their session content. For controls, each session included content representative of the e-based support provided by heart health organizations at the inception of the study [13]. In addition to the materials made available to participants assigned to the control condition, the e-counseling intervention used key components from motivational interviewing [14] and cognitive behavioral therapy [15] to promote adherence to self-care behaviors. In keeping with guidelines from motivational interviewing [14], e-counseling sessions in the early phase of the e-counseling intervention were designed to resolve ambivalence about behavior change and to help participants feel connected to a salient personal goal. Subsequent sessions provided videos, online handouts, and monitoring forms to guide and reinforce skills to sustain positive changes in targeted self-care behaviors.

As a part of the parent study, all participants were asked to complete in-person study assessments at baseline, 4-month, and 12-month follow-up. All in-person study assessments were conducted by a trained nurse or research assistant and included the collection of both questionnaire and physiological data.

### Participants

This study included 263 participants (mean age 57.5 years; 154/264, 58.3% female; 193/264, 73.1% white), who completed the baseline assessment. Inclusion criteria for the larger longitudinal study included the following: age: 35-74 years, hypertension diagnosis, baseline blood pressure measured at baseline study session: ≥140/90 (if no meds); ≥130/85 (if on meds); if on medications, an unchanged prescription for ≥2 months, and comprehension of written and oral English [13]. Exclusion criteria for the larger study included the following: diagnosis of clinically significant arrhythmia, sleep apnea, kidney disease, major psychiatric illness (eg, psychosis), alcohol or drug dependence in the previous year, institutional residence, or little to no English comprehension [13]. One participant from the larger sample was excluded from this study because of missing data on all indicators used for the analysis of psychobehavioral profiles. See Multimedia Appendix 1 for background characteristics, health behavior, and cardiovascular risk factors for total sample of parents study at baseline.

### Measures

#### Psychological Distress

Four well-established self-reported measures of psychological distress were used in this study. The Patient Health Questionnaire-9 (PHQ-9) [16] is a 9-item diagnostic screening tool that assesses symptoms of depression. The clinical interpretations of scores on this measure are as follows: 0-4=minimal depression; 5-9=mild depression; 10-14=moderate depression; 15-19=moderately severe depression; and 20-27=severe depression [16]. Both the Anxiety and Depression subscales of the Hospital Anxiety and Depression Scale (HADS) [17] also assessed symptoms of anxiety and depression, whereas the Perceived Stress Scale [18] was used to assess overall psychological distress. Only baseline measures of self-reported psychological distress were used this study [19].

#### Health Behaviors

Baseline measures of physical activity and dietary behaviors were used in this study. Physical activity was measured by calculating the 4-day number of steps recorded on a triaxial pedometer (4-day step count, LifeSource/A&D XL-18CN Activity Monitor, China) [13]. In a study of healthy adults, individuals have been classified into various lifestyle groups based on average daily step counts: (1) <5000 steps per day=sedentary lifestyle; (2) 5000-7499 steps per day=low active; (3) 7500-9999 steps per day=somewhat active; (4) ≥10,000 steps per day=active; and (5) >12,500 steps per day=highly active [20]. Moreover, according to the Canadian Hypertension Education Program (CHEP) 2016 guidelines, it is recommended that patients with hypertension engage in 30 to 60 min of moderate-intensity exercise 4 to 7 days/week, including walking, in addition to their activities of daily living [5].

Dietary behaviors were monitored by a 24-hour urinary sodium analysis (mmol/day) and the National Institute of Health/National Cancer Institute Dietary Health Questionnaire, a self-reported measure of fruit and vegetable intake, which has established validity and has been successfully adapted for a Canadian population [21]. According to the CHEP 2016 guidelines, it is recommended that patients with hypertension consume no more than 87 mmol of sodium daily and a diet high in fruits and vegetables [5].

#### Motivational Readiness to Change

Prochaska transtheoretical algorithm [22] was used as a proxy measure to assess baseline motivation to initiate or maintain self-management behaviors, including planned exercise, daily activities for active living, fruit and vegetable intake, and salt use [22]. The stages of change are conventionally defined as follows: 1=precontemplation (not ready to adhere to the target behavior in the next 6 months); 2=contemplation (ready to adhere to the target behavior in the next 6 months); 3=preparation (ready to adhere to the target behavior in the next 4 weeks); 4=action (adherence to the behavior but for less than 6 months); and 5=maintenance (adherence to the behavior for 6 months or more).

#### Physiological Measures

SBP, DBP, and PP, as well as the FRI for 10-year absolute risk of CVD [23], were assessed at baseline and 12-month endpoint. All assessments took place at the participating hospital or clinic, by a trained nurse or research assistant as noted for the REACH trial [13]. Blood pressure was assessed using a validated protocol for automated office blood pressure measurement with the BpTRU device [5]. Blood samples were taken by the hospital or clinic laboratory using conventional procedures to assess the 12-hour fasting lipoprotein cholesterol profile. The FRI was obtained from the previously noted data along with a questionnaire [23]. In addition, baseline body mass index (BMI) was calculated (kg/m^2^).

#### Program Engagement

Study completion was used as a measure of participant engagement in their assigned treatment programs. Study completion was coded as a binary measure such that 0=Incomplete, assigned to participants who did not complete the in-person 12-month study assessment, and 1=Complete, assigned to participants who completed the in-person 12-month study assessment.

### Data Analyses

#### Latent Profile Analysis

**Figure 1:**
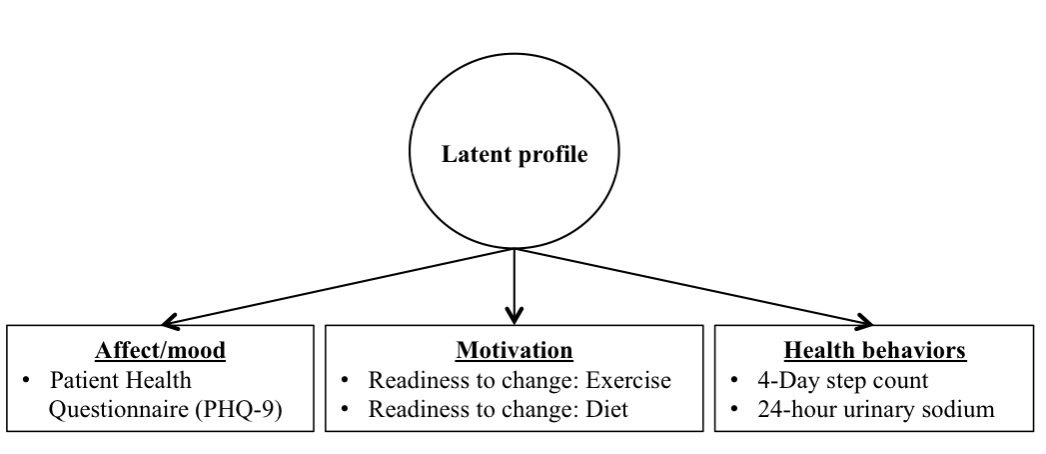
Theoretical model of latent psychobehavioral profiles of patients with hypertension.

A series of latent profile analyses (LPA) were conducted within a structural equation modeling framework to obtain psychobehavioral profiles of patients with hypertension. Psychobehavioral profiles were indicated by baseline measures of mood (PHQ-9), motivation (readiness to change: exercise and diet), and health behaviors (4-day Step Count and Urinary Sodium, see Figure 1). To determine the appropriate number of profiles, a series of models with sequentially increasing number of classes were tested for overall model fit. Each model was compared with the previous model by examining multiple indices of model fit (eg, the Bayesian Information Criterion, BIC; the sample-size adjusted BIC, ABIC; and the Lo-Mendell-Rubin test, LMR [24]). Improved model fit was indicated by smaller BIC and ABIC and a significant LMR test, and worsened model fit was evidenced by larger BIC and ABIC and nonsignificant LMR test. Patients with hypertension were then categorized into different psychobehavioral profile groups, based on saved profile classifications resulting from the LPA.

#### Analysis of Baseline and 12-Month Differences Between Profiles

Once psychobehavioral profile groups were established, a series of *t* tests and Pearson chi-square tests were performed to test the differences between psychobehavioral profile groups on baseline demographic, physiological, psychological, and behavioral variables. A series of analysis of covariance (ANCOVA) analyses were also conducted to test mean differences in baseline to 12-month assessment change in SBP, DBP, PP, and FRI between treatment groups within each of the psychobehavioral profile groups. Assessments of mean differences were made controlling for age, sex, BMI, and baseline outcome variable. Logistic regression was then used to determine differences in program engagement between profile groups.

## Results

### Latent Profile Analysis

#### Model Fit

**Figure 2:**
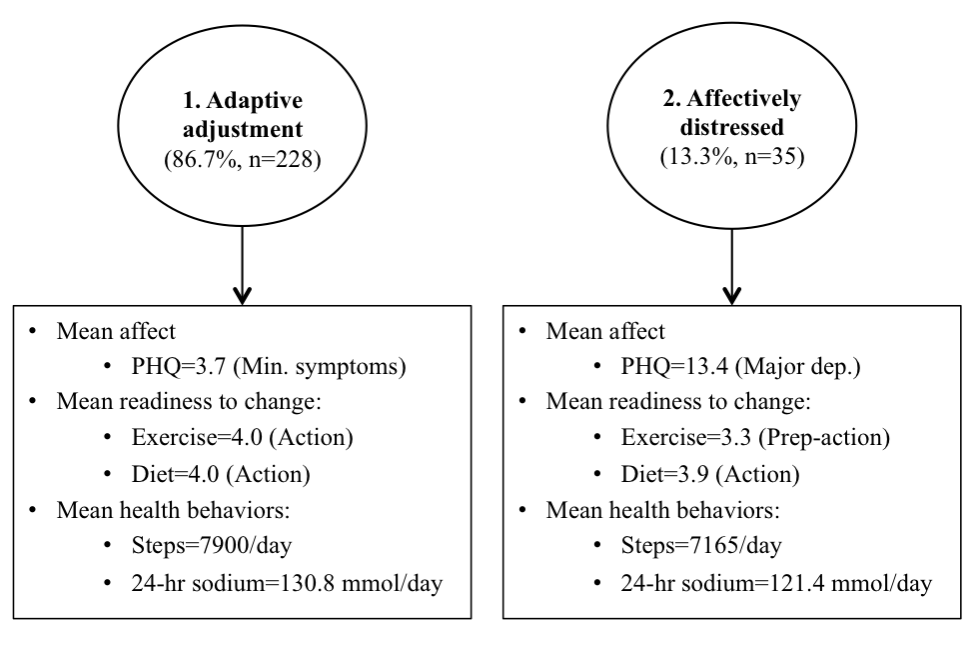
Two psychobehavioral profiles of patients with hypertension. PHQ: Patient Health Questionnaire.

A series of models with sequentially increasing number of psychobehavioral profiles of patients with hypertension were tested for overall model fit. First, indicators of psychobehavioral profiles (eg, baseline measures of mood, motivation, and health behaviors) were assessed in a 1-class LPA (BIC=10,166.92; ABIC=10,119.36). Second, a 2-class LPA using indicators of psychobehavioral profiles was tested (BIC=10,133.11; ABIC=10,006.54; LMR=65.56; *P*=.001). This analysis was followed by a 3-class LPA using these same indicators (BIC=10,116.72; ABIC=10,031.11; LMR=48.59; *P*=.59). It was determined that the 2-class solution provided the best model fit because the 3-class model of psychobehavioral profiles did not significantly improve overall model fit from the 2-class model, and the 2-class model was found to have significantly improved model fit over the 1-class model (Figure 2).

#### Psychobehavioral Profile Groups

The 2-class solution revealed 2 main psychobehavioral profiles of hypertensive patients. Most patients (228/263, 86.7%) were found to be adaptively adjusting to their hypertension diagnosis. The psychobehavioral profile of the adaptive adjustment group was marked by symptoms of depression in the minimal range of clinical severity (mean_PHQ_ 3.69, SD 0.23), relatively high motivation to adhere to guidelines for both diet and exercise (mean_readiness diet_ 3.98, SD 0.05; mean_readiness exercise_ 3.98, SD 0.07, both approaching action stage), and somewhat active engagement in physical activity (mean_steps_ 7900.38, SD 222.25). Nevertheless, on average, the adaptive adjustment group showed very poor adherence to a low-sodium diet as indicated by 24-hour urinary sodium (mean_sodium_ 130.81, SD 4.47; Figure 2).

A minority of patients (13.3%, 35/263) were classified in the second psychobehavioral profile group, which was marked by moderately elevated levels of depression, indicative of clinically significant distress (mean_PHQ_ 13.39, SD 0.83). This affectively distressed group also demonstrated relatively lower levels of motivation to exercise (mean_readiness exercise_ 3.34, SD 0.25, preparation stage) and physical activity (mean_steps_ 7165.43, SD 562.44, low active). Although this group indicated motivation to change dietary behavior indicative of individuals approaching the action stage (mean_readiness diet_ 3.85, SD 0.13), adherence to sodium intake guidelines was poor (mean_sodium_ 121.44, SD 9.74; Figure 2).

### Baseline Mean Differences Between Profile Groups

#### Demographic Differences

The *t* tests revealed a significant difference in mean baseline income between the psychobehavioral profile groups (*t*_237_=−3.73, *P*<.001), with the adaptive adjustment group having a higher average income compared with the affectively distressed group. No significant baseline differences were found between the 2 profile groups on baseline age, gender, or level of education (Table 1).

#### Physiological Differences

The *t* tests revealed a significant difference in mean baseline BMI between the psychobehavioral profile groups (*t*_261_=2.95, *P*=.003), with the adaptive adjustment group having a lower BMI (mean_BMI_ 30.63, SD 6.10), compared with the affectively distressed group (mean_BMI_ 33.97, SD 7.14). No significant baseline differences were found between the 2 profile groups on SBP, DBP, or PP (Table 1).

#### Psychological Differences

The *t* tests revealed significant differences between the psychobehavioral profile groups across several measures of psychological distress, including HADS-anxiety (*t*_249_=8.10, *P*<.001), HADS-depression (*t*_251_=11.15, *P*<.001), and Perceived Stress Scale (*t*_247_=6.66, *P*<.001). As expected, across all 3 of these measures, the adaptive adjustment group had a lower mean level of distress compared with the affectively distressed group (Table 1).

#### Behavioral Differences

The *t* tests revealed a difference in fruit and vegetable intake between the psychobehavioral profile groups that was approaching significance (*t*_241_=−1.87, *P*=.06) at baseline. The adaptive adjustment group reported, on average, higher levels of fruit and vegetable intake (mean_servings_ 8.36, SD 5.55), compared with their affectively distressed counterparts (mean_servings_ 6.43, SD 4.16; Table 1).

### Twelve-Month Treatment Effects Within Profile Groups

#### Adaptive Adjustment Group

In keeping with the therapeutic changes at 12 months reported for the REACH trial [25], ANCOVA analyses revealed significant differences in change in SBP, PP, and FRI from baseline to 12-month follow-up, (*F*_1_=5.80, *P*=.02; *F*_1_=4.27, *P*=.04; *F*_1_=5.39, *P*=.02, respectively), even when controlling for age, sex, baseline BMI, and baseline values. Adaptively adjusted participants assigned to the treatment group showed, on average, a greater decrease in change in SBP, PP, and FRI compared with their control group counterparts. Nevertheless, for adaptively adjusted participants, mean change in DBP from baseline to 12-month follow-up did not differ significantly between treatment and control groups (*F*_1_=2.28, *P*=.13; Table 2).

#### Affectively Distressed Group

ANCOVA analyses were not conducted for the affectively distressed group because of the small number of participants assigned to each group (n_control_=16, n_treatment_=4) and the wide variability in change in SBP, PP, and FRI, from baseline to 12-month follow-up (Table 3).

### Differences in 12-Month Program Engagement Between Profile Groups

Logistic regression found a significant positive effect of profile group on program engagement, as assessed by completion of the in-person 12-month study assessment (beta=0.88, SE=0.386, *P*=.02), even when controlling for age, sex, baseline SBP, and treatment group. Adaptively adjusted participants were 2.41 times more likely than the affectively distressed participants to complete the in-person study assessments, Exp(B)=2.41, regardless of age, gender, baseline SBP, or assigned treatment group.

**Table 1:** Assessing baseline outcome mean differences between profile groups. BMI: body mass index; BP: blood pressure; HADS: Hospital Anxiety and Depression Scale.

Outcome variable		Profiles		*t* test (df)	*P* value
		Adaptive adjustment (n=228)	Affectively distressed (n=35)		
**Demographics **				
	Females, n	131	23	N/A^a^	N/A
	Age, mean (SD)	57.88 (9.53)	55.51 (9.32)	−1.37 (261)	.17
	Income^b^, mean (SD)	7.46 (2.80)	5.47 (2.86)	−3.73 (237)	<.001
	Education, mean (SD)	16.22 (2.62)	15.28 (3.01)	2.03 (250)	.06
**Physiological, mean (SD)**					
	Systolic BP	141.02 (11.46)	140.17 (11.45)	−0.41 (261)	.69
	Diastolic BP	87.26 (8.67)	87.26 (8.34)	−0.001 (261)	.99
	Pulse pressure	53.76 (12.51)	52.91 (12.53)	−0.37 (261)	.71
	Framingham risk index	16.24 (10.71)	15.11 (12.03)	−0.56 (257)	.58
	BMI	30.63 (6.10)	33.97 (7.14)	2.95 (261)	.003
**Psychological, mean (SD)**					
	Patient Health Questionnaire	3.69 (.23)	13.39 (.83)	N/A	N/A
	HADS: Anxiety	5.40 (3.43)	10.59 (3.74)	8.1 (249)	<.001
	HADS: Depression	2.96 (2.64)	8.53 (3.13)	11.15 (251)	<.001
	Perceived stress	14.75 (5.04)	20.97 (4.70)	6.66 (247)	<.001
**Behavioral, mean (SD)**					
	4-day step count	7900.38 (222.25)	7165.43 (562.44)	N/A	N/A
	24-hour urinary sodium^c^	130.81 (4.47)	121.44 (9.74)	N/A	N/A
	Fruit and vegetable intake	8.36 (5.55)	6.43 (4.16)	–1.89 (241)	.06
**Motivational, mean (SD)**					
	Exercise	3.98 (0.05)	3.34 (0.25)	N/A	N/A
	Diet	3.98 (0.07)	3.85 (0.13)	N/A	N/A

^a^N/A: not applicable.

^b^Self-reported income per family (Can $), 1=≤$19,000, 10≥$100,000.

^c^89.4% (236/264) of all participants completed the baseline 24-hour urinary sodium assessment.

**Table 2:** Change in main outcomes at 12 months in adaptive adjustment group by treatment allocation. DBP: diastolic blood pressure; FRI: Framingham risk index; PP: pulse pressure; SBP: systolic blood pressure.

Adaptive adjustment	Treatment group		*F* test (df)	*P* value
	e-Counseling (n=96), mean (SD)	Control (n=80), mean (SD)		
ΔSBP	−10.51 (1.31)	−5.81 (1.44)	5.80 (1)	.02
ΔDBP	−5.15 (0.78)	−3.40 (0.86)	2.28 (1)	.13
ΔPP	−5.40 (0.90)	−2.63 (0.99)	4.27 (1)	.04
ΔFRI	−2.24 (0.64)	−0.02 (0.70)	5.39 (1)	.02

**Table 3:** Change in main outcomes at 12 months in affectively distressed group. DBP: diastolic blood pressure; FRI: Framingham risk index; PP: pulse pressure; SBP: systolic blood pressure.

Affectively distressed^a^	Treatment group	
	e-Counseling (n=4), mean (SD)	Control (n=16), mean (SD)
ΔSBP	−12.00 (8.25)	−6.63 (15.01)
ΔDBP	−11.00 (8.29)	−4.25 (8.51)
ΔPP	−1.00 (15.94)	−2.37 (11.10)
ΔFRI	−2.04 (1.34)	−0.93 (6.05)

^a^ANCOVA analyses were not conducted for the affectively distressed group because of small sample size; however, raw means and standard deviation are reported.

## Discussion

### Principal Findings

This study identified 2 baseline psychobehavioral profiles for hypertensive patients: adaptive adjustment and affectively distressed. The affectively distressed group had significantly lower income and significantly elevated baseline BMI and levels of distress (eg, anxiety, depression, and perceived stress) and engaged in the Web-based counseling program less than their adaptively adjusted counterparts. Moreover, treatment effects on SBP, PP, and FRI were statistically significant in the adaptive adjustment group but failed to reach statistical significance for DBP. These findings indicate that a large majority of patients with hypertension are likely to benefit greatly from interventions designed to provide practical support regarding adherence to lifestyle recommendations for the management of hypertension. Nevertheless, a minority of patients may also benefit from additional support to help manage psychological symptoms and associated stressors that may interfere with a patient’s ability to adhere to Web-based interventions and suggested lifestyle changes.

### Psychobehavioral Profiles

Most participants (86.7%, 228/263) in this study were found to be psychologically well-adjusted to their diagnosis and indicated relatively high motivation to engage in both healthy diet and exercise behaviors. Participants categorized in the adaptive adjustment group reported levels of motivation and physical activity within expected ranges for a cohort of patients seeking help in lifestyle behavior changes to manage their blood pressure. For example, the mean readiness for change in exercise and the somewhat active range of physical activity found in the adaptive adjustment group was comparable to a previous report of both motivation to increase physical activity and engagement in physical activity in a large sample of individuals diagnosed with CVD and/or diabetes or who were at high risk of CVD [26]. It is important to recognize that, although this group is well adjusted to their hypertension diagnosis, there is still a need for this group to engage in a comprehensive lifestyle counseling program. For instance, although this group reported high motivation for change in dietary behaviors, their urinary sodium excretions indicated difficulty in adhering to low-sodium diet recommendations [5]. Similarly, although motivation to increase exercise is relatively high in this group, average 4-day step counts indicate suboptimal level of activity [20].

A small proportion of the participants in this study (13.3%, 35/263) was identified as being affectively distressed, as their profile was marked by clinically significant elevations in depressive symptoms. The rate of clinically elevated depressive symptoms is comparable to rates of diagnosis of anxiety or depression previously reported in a large sample of hypertensive patients [27]. Although the affectively distressed group reported comparable levels of motivation for change in exercise as their adaptively adjusted counterparts, this group engaged in relatively lower amounts of physical activity. In addition, the affectively distressed group reported lower motivation to engage in dietary changes, and their urinary sodium excretion was somewhat lower than the adaptively adjusted group. Discrepancies between self-reported ratings of motivation to change and more objective health behavior indices may be an important clinical manifestation of the overall distress experienced by individuals in this psychobehavioral profiles group and may be important to address in interventions tailored for this affectively distressed group of patients with hypertension.

### Differences Between Adaptive Adjustment and Affectively Distressed Profile Groups

#### Baseline Characteristics

Examination of baseline differences between the profile groups worked to further validate the 2 psychobehavioral groups identified in this study. As would be expected, the 2 profile groups differed significantly in their baseline endorsement of psychological distress. On average, the affectively distressed group was more anxious, depressed, and stressed compared with their adaptive adjustment counterparts. The affectively distressed group also reported, on average, a lower household income compared with their adaptively adjusted counterparts. These results indicate that patients who are classified in the affectively distressed group may not only experience clinically elevated symptoms of depression but also the elevated symptoms of a wide range of other psychological symptoms and associated stressors that are also important to acknowledge and address when planning interventions for this subgroup of patients with hypertension.

Although there were no significant differences between these groups on baseline measures of SBP, DBP, or PP, the 2 groups did differ in baseline BMI. The affectively distressed group had significantly higher mean BMI than the adaptive adjustment group. The strong reciprocal association between depressive symptoms and obesity has been well established, and it has been hypothesized that multiple biological, psychosocial, and behavioral pathways likely account for this association [28]. For example, from a behavioral perspective, the adaptive adjustment group had a higher mean 4-day step count compared with the affectively distressed group. Similarly, the results of this study indicated that, on average, adaptive adjustment group may consume higher levels of fruit and vegetable compared with their affectively distressed counterparts. Because symptoms of depression often include amotivation and decreased engagement in activities, elevations in depressive symptoms can place patients with hypertension at higher risk for noncompliance to self-care recommendations. These differences in health behaviors, in addition to differences in metabolic processes, may contribute to the higher mean BMI in the affectively distressed group. It is important to note, however, that both the adaptively adjusted and affectively distressed groups had, on average, BMIs in the obese range. Because of the reciprocal relation between obesity and depression, it would be important for interventions designed for adaptively adjusted groups to provide basic psychoeducation regarding stress and coping and their potential impact on healthy lifestyle maintenance.

#### Twelve-Month Outcomes

Analysis of 12-month outcomes within the adaptive adjustment group indicated that the e-counseling program effectively reduced SBP, PP, and FRI for this profile group. Although there was no treatment effect for DBP overall, these outcomes are similar to those found in the primary outcomes paper for the larger study (unpublished data, 2018, [29]) and indicated that for most hypertensive patients, the current e-counseling program is likely effective in lowering risk for the development of CVD. Due to our limited sample size, we were unable to statistically examine these same 12-month outcomes within the affectively distressed group. It is important to note, however, that there was a pattern of greater improvements in outcome measures (SBP, DBP, PP, and FRI) for distressed patients receiving the e-counseling intervention compared with their control counterparts. This pattern of results indicates that, the current e-counseling program may be effective in reducing risk for these patients. However, because of our lack of power to assess this effect, no such conclusions can be made at this time.

#### Program Engagement

This study also found that the adaptively adjusted participants were 2.4 times more likely than the affectively distressed participants to complete both baseline and 12-month follow-up assessments. This finding is important, as it highlights a potentially significant difference in program engagement between profile groups. This finding is consistent with other studies that have found that psychological symptom severity is an important predictor of adherence to Web-based interventions [30]. Therefore, early identification of those who are significantly distressed and tailoring of interventions to address potential psychosocial barriers to program engagement is likely an important aspect of designing longitudinally effective person-centered internet-based intervention programs for patients with hypertension.

### Clinical Implications

As noted previously, psychobehavioral profiles identified in this study are important to consider from a clinical perspective when looking to implement large-scale Web-based lifestyle intervention programs for hypertensive patients, such as the program tested in the parent REACH study [13]. The psychobehavioral profiles found in this study are promising, as they show that most patients with hypertension who enroll in a Web-based behavioral counseling program are likely to be highly motivated and already on the road to effective engagement in recommended self-care behaviors. Therefore, most patients will likely benefit from programs focused on behavioral adjustments to achieve a heart-healthy lifestyle, basic psychoeducation regarding the association between stress and the maintenance of a healthy lifestyle, and strategies to maintain such changes over time.

Nevertheless, this study also identified a minority of patients who indicated that they experienced clinically significant elevations in low mood. Identification of these distressed patients is likely critical. Previous studies have indicated that patient distress or depression impairs their ability to adhere to self-care behavior and to engage in programs focused on promoting therapeutic change in lifestyle [31-33]. Our findings suggest that among patients who endorse significantly higher levels of distress, programs may prioritize supplementary self-help counseling to reinforce cognitive behavioral skills aimed at reducing psychological symptoms and managing associated stressors, before presenting strategies to maintain or adhere to recommended self-care behaviors. It is also interesting to note that a diagnosis of anxiety or depression has been found to be associated with greater health care utilization and faster blood pressure control in patients diagnosed with hypertension [27]. Such studies highlight the complexities of the pathways by which psychological symptoms ultimately influence blood pressure in patients with hypertension, and they are important to keep in mind when developing content for a wide-reaching program likely to recruit a diverse population of patients.

### Limitations and Future Directions

Although this study provides a promising new way to tailor Web-based health behavior counseling interventions for patients with hypertension, there are limitations with regard to these results. First, it is important to consider limits to the generalizability of these findings. This study sample represents a cohort of patients with hypertension who actively sought information regarding a Web-based program for self-care adherence. The relatively high levels of motivation and moderate levels of engagement in physical exercise seen in the sample may be a reflection of sampling bias introduced in the recruitment strategy. Our sample comprised health information seekers who had initially landed on the website of a public heart health organization and responded to our invitation about participating in a research project [13]. The overall population of hypertensive patients may include a wider range of psychobehavioral profiles. For example, there may be a subgroup of patients who are both highly distressed and highly unmotivated to change their lifestyle or a subgroup of patients who are highly motivated and who have been highly effective in adhering to recommended self-care behaviors. Second, this study was underpowered to detect treatment effects within the affectively distressed group, making it difficult to make any definitive conclusions regarding the effects of the e-counseling program on those participants who are most distressed on entering the program. Similarly, because of the greatly different sample sizes between the 2 profile groups, we were unable to conduct a more comprehensive analysis comparing treatment effects directly across these groups. This study was also limited in its ability to assess program engagement. Completion of the in-person 12-month study assessment was used as a proxy for program engagement, as more detailed information regarding program engagement (eg, number of emails opened or online resources accessed) was not available at the time of this study.

Future studies may aim to identify psychobehavioral profiles across a wider range of patients with hypertension to get a more accurate estimate of whether the 2 profiles reported here are replicable. Furthermore, it would be important to examine whether individuals with varying profiles respond differently to interventions aimed at promoting self-care adherence. Future randomized controlled trials may aim to oversample patients who are particularly distressed at baseline to directly examine how treatment effects may differ across varying psychobehavioral profiles. Moreover, future studies would benefit from working to eliminate in-person assessments and collecting detailed information regarding the degree to which participants engage in online programs to better define and tailor such interventions for a heterogeneous population of patients. Nevertheless, this study underscores the importance of identifying and understanding psychobehavioral profiles, as they allow for efficient evidence-based tailoring of lifestyle counseling programs for patients with hypertension.

### Conclusions

This study identified 2 latent psychobehavioral profiles for hypertensive patients based on an analysis of baseline characteristics: adaptive adjustment (86.7%, 228/263) and affectively distressed (13.3%, 35/263). Those in the affectively distressed group had significantly lower self-reported household income, elevated BMI, higher levels of distress (eg, anxiety, depression, and perceived stress) and significantly lower program adherence compared with their adaptively adjusted counterparts. The adaptively adjusted patients enrolled in an e-counseling intervention also showed significant improvements in SBP, PP, and FRI compared with their control counterparts. Accordingly, a large majority of patients with hypertension are likely to respond well to Web-based interventions designed to provide practical support regarding adherence to lifestyle recommendations for the management of hypertension. It also indicates that a minority of patients may require additional support to help manage psychological symptoms and associated stressors that may interfere with their ability to implement and adhere to suggested changes. The establishment of such psychobehavioral profiles provides an evidence-based strategy to understand the variability in patients with hypertension interested in enrolling in a Web-based intervention for lifestyle change. Such information is imperative in the development of effective person-centered Web-based interventions for a broad sample of patients with hypertension.

## Appendix

Multimedia Appendix 1 Background Characteristics, Health Behavior and Cardiovascular Risk Factors for Total Sample at Baseline.

## Abbreviations

ABIC: sample-size adjusted Bayesian information criterion
ANCOVA: analysis of covariance
BIC: Bayesian information criterion
BMI: body mass index
CHEP: Canadian Hypertension Education Program
CVD: cardiovascular disease
DBP: diastolic blood pressure
FRI: Framingham risk index
HADS: Hospital Anxiety and Depression Scale
LMR: Lo-Mendell-Rubin likelihood ratio test
LPA: latent profile analysis
PHQ-9: Patient Health Questionnaire
PP: pulse pressure
REACH: Reducing risk with E-based support for Adherence to lifestyle Change in Hypertension
SBP: systolic blood pressure
