# Supplementary material for: Psychobehavioral Profiles to Assist Tailoring of Interventions for Patients With Hypertension: Latent Profile Analysis
Source: J Med Internet Res. 2018 May 11;20(5):e149. doi: 10.2196/jmir.8757 (PMC5970280; doi:10.2196/jmir.8757)
Supplement: Multimedia Appendix 1 [file jmir_v20i5e149_app1.pdf]

Online Supplement Table 1

Supplementary Table 1: Background Characteristics, Health Behavior and Cardiovascular Risk Factors for Total Sample at Baseline

Baseline Variables	**Total Sample: N = 264**	
**Sociodemographic Features**	**n, M**	**(%), 95%CI**
Sex: % female	154	(58)
Age: years	57.6	56, 59
Body Mass Index	31.1	30, 32
Years Education	16.1	15.8, 16
Self-Reported Income*	7.2	7, 8
**Health Behavior**		
Current Smoking	24	(9.1)
4-Day Step Count†	7828	7430, 8226
Daily Fruit-Vegetable Servings‡	8.1	7.4, 8.8
**CVD Risk Factors**		
Systolic blood pressure: mmHg	141.0	140, 142
Diastolic blood pressure: mmHg	87.3	86, 88
Pulse Pressure: mmHg	53.7	52, 55
Total Cholesterol: mg/dl	195.7	191, 201
Non-HDL-Cholesterol: mg/dl	142.3	137, 147
Framingham Index:<br>10-Year Absolute CVD Risk %	15.6	14, 17

* Self-reported income per family ($CAD), 1 = ≤ $19,000, 10 ≥ $100,000.

† Step count recorded on a tri-axial accelerometer, LifeSource/A&D XL-18CN Activity Monitor

‡ Fruit and vegetable servings measured by Diet History Questionnaire [21].
